# Acupuncture-Evoked Response in Somatosensory and Prefrontal Cortices Predicts Immediate Pain Reduction in Carpal Tunnel Syndrome

**DOI:** 10.1155/2013/795906

**Published:** 2013-06-17

**Authors:** Yumi Maeda, Norman Kettner, Jeungchan Lee, Jieun Kim, Stephen Cina, Cristina Malatesta, Jessica Gerber, Claire McManus, Jaehyun Im, Alexandra Libby, Pia Mezzacappa, Leslie R. Morse, Kyungmo Park, Joseph Audette, Vitaly Napadow

**Affiliations:** ^1^Athinoula A. Martinos Center for Biomedical Imaging, Department of Radiology, Massachusetts General Hospital, No. 2301 149 Thirteenth Street, Charlestown, MA 02129, USA; ^2^Department of Radiology, Logan College of Chiropractic/University Programs, Chesterfield, MO 63017, USA; ^3^Department of Biomedical Engineering, Kyung Hee University, Yongin, Gyeonggido 446-701, Republic of Korea; ^4^Department of Physical Medicine and Rehabilitation, Spaulding Rehabilitation Hospital, Medford, MA 02155, USA; ^5^Department of Physical Medicine and Rehabilitation, Harvard Medical School, Spaulding Rehabilitation Hospital, Boston, MA 02114, USA; ^6^Department of Pain Medicine, Harvard Vanguard Medical Associates, Atrium Health, Boston, MA 02215, USA

## Abstract

The linkage between brain response to acupuncture and subsequent analgesia remains poorly understood. Our aim was to evaluate this linkage in chronic pain patients with carpal tunnel syndrome (CTS). Brain response to electroacupuncture (EA) was evaluated with functional MRI. Subjects were randomized to 3 groups: (1) EA applied at local acupoints on the affected wrist (PC-7 to TW-5), (2) EA at distal acupoints (contralateral ankle, SP-6 to LV-4), and (3) sham EA at nonacupoint locations on the affected wrist. Symptom ratings were evaluated prior to and following the scan. Subjects in the local and distal groups reported reduced pain. Verum EA produced greater reduction of paresthesia compared to sham. Compared to sham EA, local EA produced greater activation in insula and S2 and greater deactivation in ipsilateral S1, while distal EA produced greater activation in S2 and deactivation in posterior cingulate cortex. Brain response to distal EA in prefrontal cortex (PFC) and brain response to verum EA in S1, SMA, and PFC were correlated with pain reduction following stimulation. Thus, while greater activation to verum acupuncture in these regions may predict subsequent analgesia, PFC activation may specifically mediate reduced pain when stimulating distal acupoints.

## 1. Introduction 

Acupuncture, a component of traditional Chinese medicine, has been commonly applied to alleviate symptoms of patients with chronic pain [1]. Carpal tunnel syndrome (CTS) is mainly driven by partial deafferentation secondary to compression of the median nerve within the carpal tunnel [2]. CTS clinically manifests as slowing of median nerve conduction velocity, local pain, and paresthesia. Recent randomized controlled trials (RCT) for CTS have shown that acupuncture produced significant improvement in symptoms, with effects similar to steroid treatment [3] and night splinting [4]. A recent RCT demonstrated that acupuncture also reduced CTS symptoms significantly greater than placebo [5]. 

Noninvasive brain imaging techniques, such as functional MRI (fMRI), have offered an unprecedented window into how the human brain responds to acupuncture needle stimulation [6, 7]. However, very few of these studies have been performed in chronic patient populations, and even fewer have evaluated how brain response to acupuncture relates to the alleviation of clinical symptoms or even evoked-pain ratings. Zhang et al. demonstrated that fMRI response to transcutaneous electrical acupoint stimulation in brain areas such as secondary somatosensory cortex (S2), insula, and primary motor cortex (M1) was associated with reduced heat pain ratings in healthy adults [8]. More recently, Yang et al. found that acupuncture increased metabolism in regions including the prefrontal cortex (PFC), insula, and cingulate, while also decreasing pain levels in acute migraine patients in a positron emission tomography (PET) study [9]. Harris et al. used PET with ^11^C-carfentanil in fibromyalgia patients and found that long-term increases in resting mu-opioid receptor binding in regions including insula, cingulate, and basal ganglia following 4 weeks of acupuncture correlated with decreased clinical pain levels over this same time period [10]. In CTS patients, compared to healthy adults, manual acupuncture needling has been found to produce more robust fMRI response in several brain areas including insula, cingulate, S1, and PFC, when controlling for the effects of sham (noninserted cutaneous tactile) acupuncture [11]. Enhanced processing in S1 and PFC was particularly interesting, given the fact that these patients demonstrated altered somatosensory-stimulation-evoked brain response in these areas [12] and that S1 activity was specifically modulated by five weeks of acupuncture treatment [13]. Unfortunately, changes in clinical pain were not evaluated in this study. Thus, the association between the brain circuitry processing acupuncture stimulation and postacupuncture clinical outcomes such as pain reduction is currently unknown. 

In this cross-sectional study, CTS subjects were randomized to 3 groups: (1) EA applied at local acupoints on the affected wrist (PC-7 to TW-5), (2) EA at distal acupoints (contralateral ankle, SP-6 to LV-4), and (3) sham EA at nonacupoint locations on the affected wrist. In addition to fMRI data acquired during EA, we also evaluated changes in clinical symptoms following acupuncture and correlated fMRI response to EA with changes in symptoms. We hypothesized that both local and distal EA would produce greater symptom reduction compared to sham acupuncture. Furthermore, we hypothesized that the magnitude of symptom reduction would correlate with activation in brain regions previously associated with somatosensory, affective, and cognitive processing of pain and paresthesia in CTS subjects.

## 2. Methods

### 2.1. Subjects

Subjects, aged 20 to 60, with a 3-month or greater history of pain and/or paresthesia in median-nerve-innervated areas were enrolled. All subjects were examined for eligibility by a physiatrist at Spaulding Rehabilitation Hospital, which included a physical exam for Phalen's maneuver [14] and Durkan's sign [15] and testing of median and ulnar sensory nerve conduction (NCS: Cadwell Sierra EMG/NCS Device, Kennewick, WA). NCS inclusion criteria consisted of median nerve sensory latency greater than 3.7 ms or median nerve sensory latency greater than 0.5 ms compared to ulnar nerve. Exclusion criteria consisted of contraindications to MRI, history of diabetes mellitus, cardiovascular, respiratory, or neurological illnesses, rheumatoid arthritis, wrist fracture with direct trauma to median nerve, current usage of prescriptive opioid medication, thenar atrophy, previous acupuncture treatment (manual, EA, and TENS) for CTS, nerve entrapment other than median nerve, cervical radiculopathy or myelopathy, generalized peripheral neuropathy, blood dyscrasia or coagulopathy or current use of anticoagulation therapy. History of axis I psychiatric diagnosis (substance use disorder, psychotic disorder, or bipolar disorder), and use of psychotropic medications were also exclusions for this study. Chronic symptomatology for all eligible subjects was evaluated using the Boston Carpal Tunnel Syndrome Questionnaire (BCTSQ) [16].

A total of 59 CTS subjects (49.1 ± 9.8 years old, mean ± SD, 49 Female) were enrolled in this study. For the subjects who had diagnosed bilateral CTS, the more affected hand was determined as the test hand. CTS subjects were randomized to one of the three study arms: (1) local verum EA (*n* = 22, 17F, 14 right hand affected), (2) distal verum EA (*n* = 18, 14F, 13R), and (3) sham EA (*n* = 19, 18F, 11R). All study protocols were approved by the Massachusetts General Hospital and Partners Human Research Committee. Written informed consent was obtained from all subjects. 

### 2.2. Acupuncture Procedure

**Figure 1:**
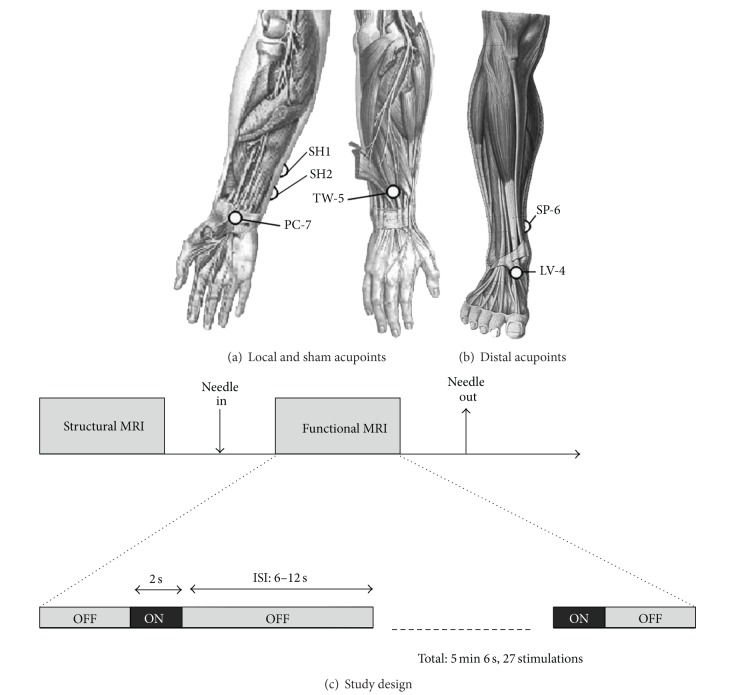
Acupoints and schematic scan session. Verum EA was performed at both (a) local (PC7 to TW5) and (b) distal (SP6 to LV4) acupoints. Sham EA used noninsertive needles placed over sham points, SH1 and SH2. (c) Our fMRI event-related study design for acupuncture stimulation.

For local verum EA, MRI-compatible titanium needles (0.2 mm in diameter, 35–50 mm in length, DongBang Acupuncture Inc. Boryeong, Korea) were inserted and *deqi* sensation elicited at acupoints PC7 (pericardium 7, 1st wrist crease) and TW5 (triple-warmer 5, dorsal aspect of forearm), local to the more affected hand (Figure 1(a)). PC-7 was chosen because it is close to the CTS lesion, and this set of points was found to reduce pain and paresthesia in our previous study [13]. 

For distal verum EA, MRI-compatible titanium needles were inserted and *deqi* sensation elicited at acupoints SP6 (spleen 6, medial aspect of lower leg) and LV4 (liver 4, anterior aspect of the ankle) at the ankle on the contralateral side to the more affected hand (Figure 1(b)). Distal acupoints LV4 and SP6 were chosen based on mirror point methods common in acupuncture practice, where acupoints on the leg/ankle can be used to treat symptoms on the opposite arm/wrist [17].

For sham EA, MRI-compatible blunt-tipped acupuncture needles were placed with a single tap but not inserted percutaneously, over sham points, SH1 (2 cun, or roughly 2-3 cm, distal and slightly volar to acupoint SI-7, which is 5 cun proximal to ulnar edge of the transverse wrist crease, ulnar forearm) and SH2 (1 cun distal and slightly volar to SI-7), on the more affected hand (Figure 1(a)). 

For all 3 groups, needles were connected to a constant current electroacupuncture (EA) device (HANS LH202H, Neuroscience Research Center, Peking University, Beijing, China). A licensed acupuncturist trained to place and stimulate acupuncture needles in the scanner performed these procedures. For verum EA, current stimulation frequency was set to 2 Hz, while the current intensity was set just prior to the functional scan. The acupuncturist gradually increased the current intensity until the subject felt a moderately strong but not painful sensation. For sham EA, electrodes were attached to the needles, but no electrical current was passed. Subjects were instructed that the current intensity was set to a predetermined level and that they may or may not feel any sensation at the needle sites. All subjects were instructed to close their eyes and focus on the acupuncture stimulation while remaining as still as possible. 

### 2.3. Data Acquisition

All imaging data were acquired on a 3.0T Siemens Trio (Siemens Medical, Erlangen, Germany) equipped with 32-channel head coil. Structural imaging data were acquired with a multiecho MPRAGE T1-weighted pulse sequence (TR = 2530 ms, TE1/TE2 = 1.64/30.0 ms, TI = 1200 ms, flip angle = 7°, FOV = 256 × 256, slices = 176, sagittal acquisition, spatial resolution = 1 × 1 × 1 mm^3^). 

Functional imaging (fMRI) data were acquired using a gradient echo BOLD T2*-weighted pulse sequence (TR/TE = 2000/30 ms, FOV = 200 × 200 mm, 32 axial slices parallel to anterior/posterior commissural plane, voxel size = 3.125 × 3.125 × 3.6 mm, flip angle = 90°). Subjects lay supine in the scanner with earplugs to attenuate acoustic gradient switching noise. For verum EA, stimulation was performed using an event-related design similar to our previously published approach for manual acupuncture [18] (2-second stimulation events with randomized ISI, 6–12 seconds, and total scan time 5 minutes and 6 seconds, Figure 1(c)). For sham EA, procedures were identical, but no electricity was passed through the needles.

Symptoms were assessed using a 0–10 VAS scale for both pain and paresthesia (tingling) at the hand/wrist. The scale ranged from none (0) to unbearable (10) and was administered at the beginning and the end of the MRI session. In addition, subjects were asked to rate the intensity of acupuncture-evoked sensations after the scan session using the MGH Acupuncture Sensation Scale (MASS) instrument [19]. 

### 2.4. Data Analysis

Statistical analyses for behavioral and clinical data were performed with SPSS (SPSS version 10.0.7, Chicago, Il). Median sensory nerve velocities and motor nerve latencies were compared with those of the ulnar nerve in all CTS subjects using a Student's *t*-test, significant at *P* < 0.05. 

Changes in VAS scores for pain and paresthesia were compared between groups using a mixed model ANOVA with interaction of Group (local, distal, and sham) × Time (pre and post). Student's *t*-test was used to compare change in VAS scores between verum (combined local and distal) and sham groups. EA current intensities were compared between local and distal acupoint groups using a Student's *t*-test significant at *P* < 0.05. A one-way ANOVA was performed in order to compare evoked acupuncture sensations, as well as the MASS Index (a composite metric of *deqi* sensation) between groups. Post hoc testing was performed with the Tukey test.

FMRI data were preprocessed using the FMRIB software Library (FSL v.4.1), Freesurfer (v.5.1.), and AFNI (v.2.). FMRI data were coregistered with each subject's structural MRI data using boundary-based registration (BB registration, Freesurfer [20]). Preprocessing included slice timing correction, motion correction, high pass filtering with a cut-off period of 50 sec, and spatial smoothing with Gaussian kernel at FWHM = 5 mm (Feat, FSL). Preprocessed fMRI data were then analyzed using a general linear model for all subjects (Feat, FSL), with the explanatory variable set by the event-related design. The resultant parameter estimates and variances from all subjects were transformed to standard MNI space (FNIRT, FSL) in order to perform nonflipped (compare with below) group analyses. Registration was ensured by visualization (AFNI, afni). 

In order to better assess brain response for structures known to be lateralized relative to somatosensory input (i.e., S1 and M1, and thalamus), parameter estimates and variances of subjects whose more affected hand was the left hand (and thus experienced local EA on the left hand or distal EA on the right ankle) also had their fMRI parameter estimates flipped across the midsagittal plane before passing them up to the flipped group analysis. Therefore, a total of 21 fMRI datasets (local: 8, distal: 5, sham: 8) were analyzed by flipping across the midsagittal plane.

Group maps were calculated using a mixed effects statistical model (FLAME, Feat, FSL). Difference maps were calculated with an ANOVA using a mixed effect model (FLAME, Feat, FSL). As we found no differences in brain response between local and distal groups, a combined group map for “verum EA” was also calculated in order to increase statistical power and for future testing. Whole brain regression analysis was performed for local, distal, and sham groups, as well as the combined verum group in order to identify brain regions associated with symptom reduction. For the regression analysis with symptom reduction, changes in VAS pain and paresthesia scores (post and pre) were demeaned and added as explanatory variable to the model. All statistical maps were thresholded with cluster forming threshold at *z* = 2.3 (voxel wise threshold *P* < 0.01), and cluster corrected for multiple comparisons at *P* < 0.05 [21]. 

## 3. Results

### 3.1. Demographic and Clinical Features

Bilateral CTS was diagnosed in 44/59 (75%) subjects, while unilateral CTS was diagnosed in 15/59 (25%) subjects. The more affected hand was the right hand in 38/59 (64.4%) subjects and the left hand in 21/59 (35.6%) subjects. Pain was the more severe symptom in 11/59 (18.3%) subjects; paresthesia was more severe in 37/59 (63.3%) subjects, and pain and paresthesia were observed with equal severity in 11/59 (18.3%) subjects. BCTSQ assessment of symptoms on the scale 1 to 5 demonstrated that pain and paresthesia were moderate (2.6 ± 0.9, 2.9 ± 0.8, mean ± S.D., resp.). Pain and paresthesia ratings were positively correlated (*r* = 0.68, *P* < 0.001); that is, those subjects with greater pain also reported greater paresthesia. Self-reported symptom duration was 9.0 ± 8.8 years (mean ± S.D.) and was positively correlated with subjects' age (*r* = 0.39, *P* < 0.01). A significant correlation was found between VAS pain and paresthesia scores before acupuncture (pre; *r* = 0.68, *P* < 0.001). In addition, VAS pain scores at baseline were significantly correlated with the BCTSQ pain score (*r* = 0.60, *P* < 0.001). However, VAS paresthesia scores were not significantly correlated with BCTSQ paresthesia scores (*r* = 0.25, *P* = 0.053).

Phalen's test was positive on the right hand in 39/59 (66.1%) subjects and on the left hand in 34/59 (57.6%) subjects. Durkan's test was positive on the right hand in 26/59 (44.0%) subjects and on the left hand in 24/59 (40.7%) subjects. For the study hand, Phalen's test was positive in 51/59 (86.4%) subjects, while Durkan's test was positive in 38/59 (64.4%) subjects. Median nerve sensory velocities were significantly slower compared to ulnar nerve sensory velocities (median: 37.9 ± 6.9 m/s, ulnar: 55.6 ± 6.7, mean ± SD, *P* < 0.0001). Furthermore, median nerve motor latencies were significantly longer compared to ulnar nerve motor latencies (median: 5.0 ± 1.3 ms, ulnar: 2.9 ± 0.3, mean ± S.D., *P* < 0.0001). 

The regular usage of night splints was reported in 33/59 (56.7%) subjects. Subjects' occupational status could be described as “full-time work” in 38/59 (64.4%) subjects and part-time in 8/59 (13.6%) subjects. Mean body mass index (BMI) was 29.0 ± 5.1 (mean ± SD).

### 3.2. Symptom Change following EA

**Figure 2:**
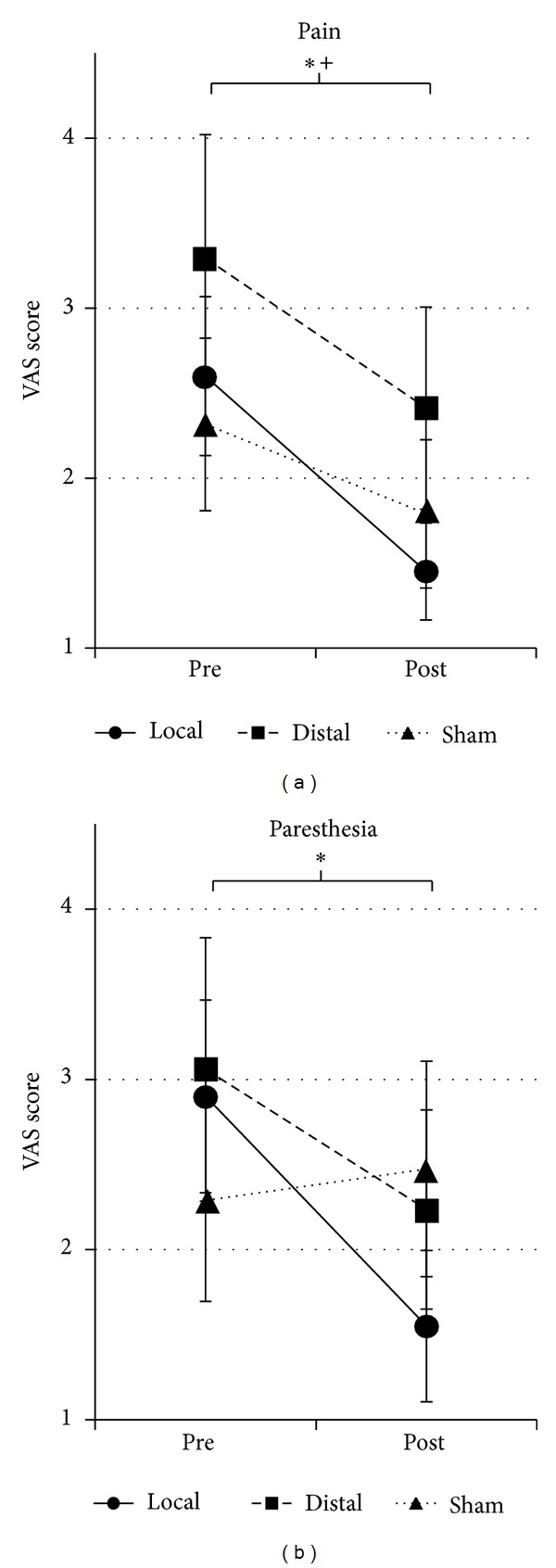
Symptom rating before and after acupuncture. A significant main effect of Time (pre versus post) was found for pain and paresthesia VAS score (*F*_1,56_ = 19.3, *P* < 0.0001; *F*_1,56_ = 5.2, *P* < 0.03, resp.), indicating reduced pain and paresthesia after acupuncture. Post hoc testing found that local EA reduced pain and paresthesia (*P < 0.01) while distal EA reduced pain (^+^ *P* < 0.05). Error bars indicate standard error of the mean.

We found a significant main effect of Time (pre versus post) for VAS score in pain and paresthesia (*F*_1,56_ = 19.3, *P* < 0.0001, *F*_1,56_ = 5.2, *P* < 0.03, resp., Figure 2). There was neither a significant main effect of Group (*P* > 0.7, *P* > 0.9, resp.) nor interaction of Time × Group for either pain or paresthesia (*P* > 0.4, *P* > 0.1, resp.). Post hoc testing revealed that VAS scores for pain showed significant reductions for local and distal groups but not for the sham group (local: −1.2 ± 1.5, *P* < 0.005; distal: −1.2 ± 2.2, *P* < 0.05; sham: −0.5 ± 1.4, *P* = 0.12; mean ± SD). VAS scores for paresthesia showed significant reductions for the local group but not for the distal or sham groups (local: −1.3 ± 1.6, *P* < 0.001; distal: −1.1 ± 2.1, *P* = 0.055; sham: 0.2 ± 3.5, *P* = 0.82; mean ± SD). Also, the combined verum (local and distal) group produced a greater reduction in VAS scores for paresthesia compared to the sham group (verum: −1.2 ± 1.8, sham: 0.2 ± 3.5, mean ± S.D., *P* < 0.05). A statistically significant reduction for verum compared to sham acupuncture was not seen in VAS scores for pain (verum: −1.2 ± 1.8, sham: −0.5 ± 1.4, mean ± SD, *P* > 0.1). 

We also found that change in VAS pain scores correlated with change in VAS paresthesia scores for both the local (*r* = 0.44, *P* < 0.04) and sham groups (*r* = 0.77, *P* < 0.001) but not for the distal group (*r* = 0.35, *P* = 0.14). 

### 3.3. Electroacupuncture Current Intensity and EA-Evoked Sensations

**Figure 3:**
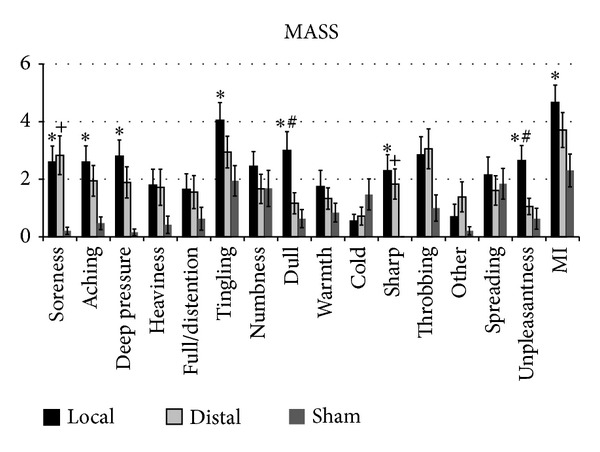
Acupuncture sensations. A significant difference between local and sham EA was found for soreness, aching, deep pressure, tingling, sharp pain, unpleasantness, and MI (*P < 0.05). A significant difference between distal and sham EA was found for soreness and sharp pain (^+^ *P* < 0.05). A significant difference between local and distal EA was found for dull pain and unpleasantness (^#^ *P* < 0.05). Error bars indicate standard error of the mean.

EA current intensity did not differ between local and distal groups (local: 1.6 ± 1.0 mA, distal: 2.0 ± 0.9, mean ± S.D., *P* > 0.3). For acupuncture-evoked sensation, an ANOVA demonstrated a significant main effect of Group for Mass index (MI, *F*_2,56_ = 6.5, *P* < 0.003, Figure 3). Post hoc testing revealed that the local group produced greater MI scores compared to the sham group (*P* < 0.02, local: 4.6 ± 2.1, distal: 3.4 ± 1.8, sham: 2.3 ± 2.0, mean ± S.D.). In regard to individual MASS sensations, a significant main effect of Group was also detected for soreness (*F*_2,56_ = 8.1, *P* = 0.001), aching (*F*_2,56_ = 5.5, *P* = 0.006), deep pressure (*F*_2,56_ = 8.7, *P* = 0.001), tingling (*F*_2,56_ = 3.8, *P* = 0.03), dull pain (*F*_2,56_ = 6.9, *P* = 0.002), sharp pain (*F*_2,56_ = 7.6, *P* = 0.001), and unpleasantness (*F*_2,56_ = 6.9, *P* = 0.003) (Figure 3). Post hoc testing revealed that the local group produced greater sensation compared to the sham group for soreness, aching, deep pressure, tingling, sharp pain, spreading, and unpleasantness (*P* < 0.05). Also, the distal group produced greater sensation compared to sham acupuncture for soreness and sharp pain (*P* < 0.05), while the local group produced greater sensation compared to the distal group for dull pain and unpleasantness (*P* < 0.05, Figure 3). EA current intensity was correlated with numbness for the local group (*r* = 0.52, *P* = 0.013). EA current intensity was correlated with soreness (*r* = 0.47, *P* = 0.048) and cold (*r* = 0.50, *P* = 0.035) for the distal group. In addition, Mass index was not correlated with pain reduction (local: *r* = −0.09, distal: *r* = 0.18, sham: *r* = 0.29) or paresthesia reduction (local: *r* = 0.29, distal: *r* = −0.32, sham: *r* = 0.44) in any group (*P* > 0.05). EA current intensity was not correlated with pain reduction (local: *r* = −0.35, distal: *r* = 0.04) or paresthesia reduction (local: *r* = 0.04, distal: *r* = 0.25) in either the local or distal group (*P* > 0.05). 

### 3.4. fMRI during Acupuncture

**Figure 4:**
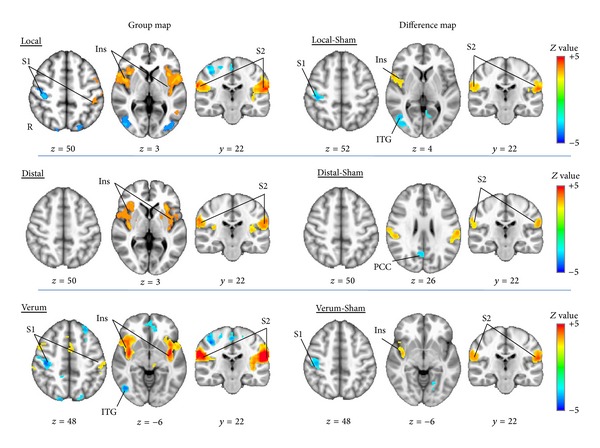
Group and difference maps of brain response during acupuncture. Local EA produced activation in contralateral primary somatosensory cortex (S1) and bilateral insulae and secondary somatosensory cortex (S2) and deactivation in ipsilateral S1. Distal EA produced activation in bilateral insula and S2. Verum EA (combined local and distal) produced activation in left S1 and bilateral insulae and secondary somatosensory cortex (S2) and deactivation in the medial prefrontal cortex (PFC) and right S1. Compared to sham EA, local EA produced greater activation in right insula and bilateral S2 and while greater deactivation in ipsilateral S1 and inferior temporal gyrus (ITG). Distal EA produced greater activation in bilateral S2 and greater deactivation in PCC. Verum EA produced greater activation in right insula and bilateral S2 and greater deactivation in right S1. Note: S1 was analyzed using a midsagittal plane flipped analysis (as S1 is known to be lateralized in activity relative to the stimulated side), while other regions were analyzed using a more conventional nonflipped analysis. All coordinates are in MNI space.

Local EA produced activation in bilateral insulae, secondary somatosensory cortex (S2), superior temporal gyrus (STG), and contralateral postcentral gyrus (S1), with deactivation in cuneus and ipsilateral S1 (Table 1, Figure 4). Distal EA produced activation in bilateral insulae, S2, and premotor cortex (PMC), while no deactivation was found (Figure 4). Sham EA showed no significant fMRI response (Table 1). There were no significant differences in brain response between local and distal groups (Table 1); hence we also calculated a combined verum (local and distal) EA group map, which showed activation in bilateral S2, premotor (PMC), supramarginal (SMG), and superior temporal (STG) gyri, as well as anterior and posterior insulae (a.Ins, p.Ins) and thalamus. Activation was also noted in right presupplementary motor area (pSMA) and middle and inferior frontal gyri (MFG, IFG) (Table 1, Figure 4). Verum EA also produced deactivation in bilateral occipital gyrus, right medial prefrontal cortex (PFC), inferior temporal gyrus (ITG), and S1 (Table 1, Figure 4).

In comparison to fMRI response for the sham group, local EA produced greater activation in bilateral S2 and right frontal insular cortex (FIC), while greater deactivation was found in right inferior temporal gyrus (ITG) (Table 2, Figure 4). Distal EA produced greater activation in bilateral S2 and deactivation in posterior cingulate cortex (PCC). Combined (local and distal) verum EA produced greater activation in bilateral S2 and insula and deactivation in PCC, precuneus, and right S1. 

**Figure 5:**
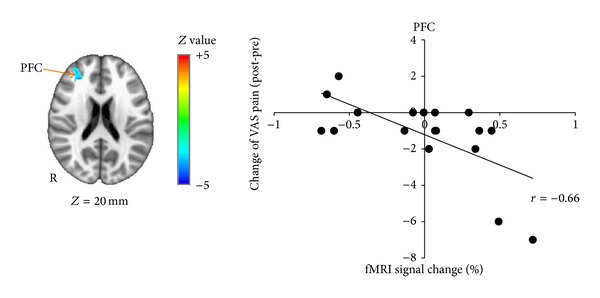
Brain response in right prefrontal cortex correlated with pain reduction in distal group. Brain response in right prefrontal cortex (PFC) was negatively correlated with change in VAS pain score (post-pre) in distal group. Percent signal change in rPFC was extracted from the peak voxel and plotted with change of VAS pain score (post-pre).

**Figure 6:**
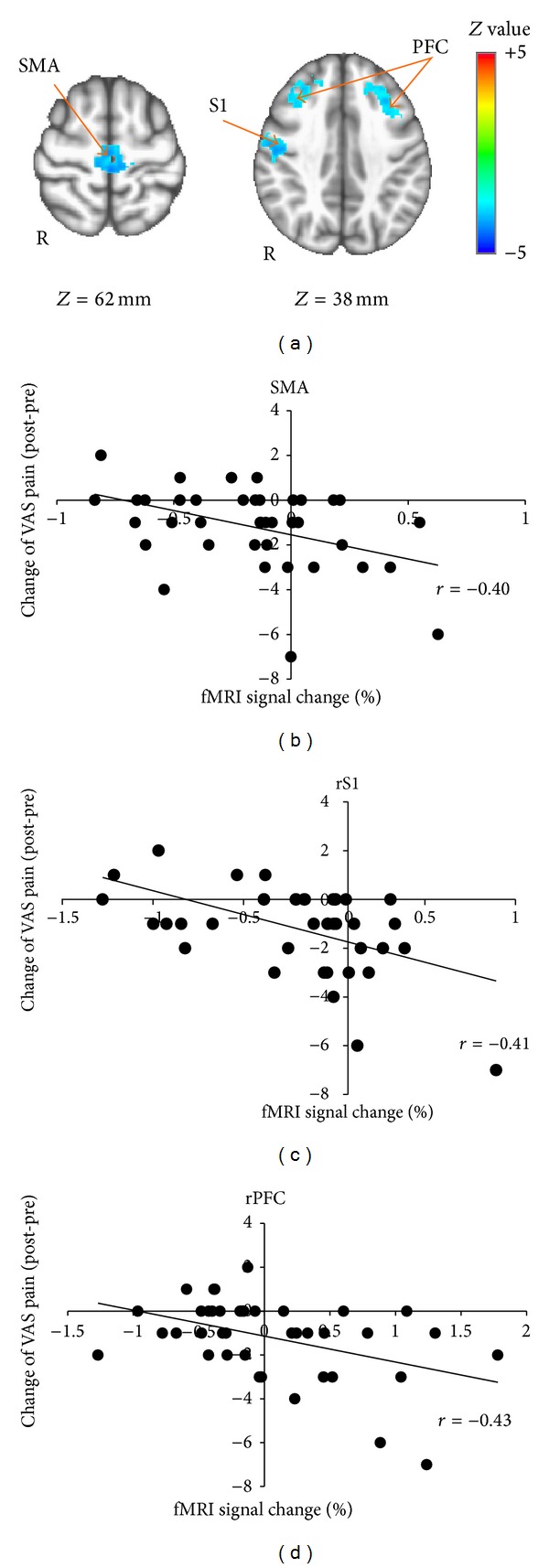
Brain response in bilateral SMA, PFC, and right S1 correlated with pain reduction in verum group. Brain response in bilateral SMA, PFC, and right S1 were negatively correlated with changes in VAS pain score (post-pre). fMRI percent signal change in SMA, PFC, and right S1 were extracted from the peak voxel and plotted with change of VAS pain score.

Whole brain regression analysis for change in VAS pain score in the distal group revealed a significant correlation in right prefrontal cortex (PFC). Thus, greater activation in PFC was associated with greater pain reduction following distal EA (Table 3, Figure 5). No significant correlations were found for either local or sham groups. For the combined verum EA group, a significant negative correlation was found between changes in VAS pain scores and brain activity in right S1 and bilateral supplemental motor area (SMA) and prefrontal cortex (PFC) (Table 3, Figure 6). Thus, greater activation in S1, SMA, and PFC was associated with greater pain reduction following verum EA.

## 4. Discussion 

This study investigated how brain response to EA was associated with symptom reduction following stimulation in CTS subjects. Our main finding was that pain was reduced following verum (both local and distal) EA and that greater brain activation in PFC, SMA, and S1 in response to verum EA was associated with more pronounced pain reduction. Thus, greater activation in these regions may be a biomarker for immediate analgesia following EA. 

Brain response to verum EA produced activation in several regions including dorsolateral prefrontal cortex (dlPFC), pre-SMA (pSMA), S1, bilateral S2, and insula, with the latter two regions also showing greater activation compared to sham EA. Deactivation was noted in ipsilateral S1 and default mode network areas such as the medial prefrontal cortex and lateral temporal cortex, also with evidence of greater deactivation compared to sham EA. These findings are consistent with multiple previous acupuncture fMRI studies [6, 7], which support the veracity of the event-related fMRI data used to correlate with clinical outcomes in this study. 

Similar to our findings, linking dorsolateral PFC (dlPFC) activation with pain reduction following EA, Yang et al. used FDG PET and found that acupuncture produced increased brain metabolism in the middle frontal gyrus (a subregion of the dlPFC) and concomitant pain reduction in acute migraine patients [9]. The prefrontal cortex is known to modulate pain [22]. Chronic pain patients demonstrated reduced dlPFC gray matter volume [23], while transcranial magnetic stimulation to this region reduced placebo analgesia [24]. Moreover, recent studies have suggested that PFC dopamine levels mediate pain sensitivity [25]. As EA has been shown to modulate fMRI activity in dopaminergic source regions (i.e., substantia nigra) in a time-dependent manner [26], analgesia related with PFC response to EA in CTS subjects may prove to be mediated by this neuromodulatory catecholamine.

PFC response may also differentiate the underlying analgesic mechanisms for local versus distal EA. Both local and distal EA reduced pain, and we found no differences in brain response between these groups. However, this result does not necessarily suggest that there is no acupoint specificity for brain-linked mediators of acupuncture analgesia in CTS. For instance, greater PFC activation to distal, but not local EA, was associated with greater pain reduction. The tissue at local acupoints (i.e., PC-7) is adjacent to the carpal tunnel, which is known to be perturbed by the increased pressure, fibrosis, swelling, and various biochemical changes within the carpal tunnel [27], while the tissue at distal acupoints is not. Thus, the mechanism by which local EA reduces pain may involve changes in local signaling from the wrist lesion (bottom, up), while the mechanism by which distal EA reduces pain may instead involve changes in brain processing (top, down), particularly in the PFC. Interestingly, multiple previous studies have found that PFC activity supports placebo [24, 28] and expectation-mediated analgesia [29]. Future studies should further explore how PFC activity relates to analgesia for different forms of acupuncture and how this is similar to or differs from contextually-mediated analgesic phenomena. 

Brain response to verum EA in right S1 was also correlated with pain reduction. We found that greater activation in this region was associated with reduced pain, while more pronounced deactivation was associated with worsening pain following verum EA. The somatotopy of this cluster is consistent with the hand area, which would be ipsilateral for the local group and contralateral (but well outside the leg area) for the distal group. In general, S1 regions outside of the contralateral somatotopic representation for somatosensory stimulated body areas are deactivated [30], and this is clear in the scatterplot for our data as well deactivation is noted in this right S1 cluster for most subjects. Interhemispheric communication modulates subcortical relay of sensory information [31]. Therefore, the balance of left and right S1 activity may modulate the amount of sensory input, which might serve to diminish the spontaneous afferent signal (e.g., pain, paresthesia) from the affected hands in our study. 

SMA activation was also correlated with pain reduction following verum EA. SMA is a cortical region that modulates communication between the somatosensory and motor systems and has been shown to be activated by painful stimulation in fMRI [32] and involved in pain control [33]. Greater activation following verum EA may reflect greater transfer of EA-induced somatosensory inputs to the motor system [34], fostering a more normalized sensorimotor communication, compared to the sporadic afference coming from diffuse paresthesia. Thus, EA (whether local or distal) may also reduce pain by supplying regulated somatosensory input to the brain. 

While verum acupuncture was found to reduce paresthesia significantly more than sham acupuncture, the same was not true for pain. Firstly, these results suggest that compared to pain, paresthesia may be less susceptible to modulation by sham acupuncture. In fact, paresthesia is a hallmark of CTS and other peripheral neuropathic pain disorders stemming from compression of the nerve trunk, and may be more dependent on peripheral factors such as hand positioning and temperature. Our data suggest that reduction of paresthesia is dependent on therapies with significant somatosensory afference, such as verum EA, regardless of whether this afference comes from the site of the lesion. Perception of paresthesia in CTS may be less centralized compared to pain and may thus be less amenable to placebo effects. However, we should also note that controversy regarding the use of sham acupuncture as a control for verum acupuncture exists, as sham procedures are not physiologically inert [35, 36]. In fact, sham acupuncture has been shown to reduce aversive symptom more readily than a placebo pill [37]. Interestingly, while analgesic outcomes may be similar between verum and sham EA, the brain mechanisms supporting this analgesia may differ substantially. In our study, brain response was more profound for verum compared to sham acupuncture, and activity in specific regions in response to verum EA was correlated with pain reductions. These results further support the growing evidence [10] that brain response may serve as an objective marker that differentiates verum and sham acupuncture more readily than subjective pain report does.

Several limitations to our study should be noted. Although brain response to verum or distal EA showed significant correlation with short-term pain reduction, we did not have enough statistical power to show significant correlations between brain response to local or sham EA alone and short-term pain reduction. In addition, our study examined brain response to EA stimulation associated with short-term clinical outcomes, and future studies should extend our analysis to longer-term outcomes. Future studies also should apply alternative fMRI approaches, such as functional connectivity [38], to evaluate how brain connectivity response to both verum and sham acupuncture mediates analgesia in CTS. 

In conclusion, EA stimulation at both local and distal acupoints reduced pain, and greater brain activation in PFC, SMA, and S1 was associated with more pronounced pain reduction. Thus, greater PFC, SMA, and S1 activation may support acupuncture analgesia. Brain response in PFC, SMA, and S1 could serve as predictive biomarkers to identify patients more likely to benefit from acupuncture therapy.

## Figures and Tables

**Table 1:** Brain response to electroacupuncture.

Region	Side	Cluster size	*P* value	max_*z*		MNI (mm)	
					*X*	*Y*	*Z*
*Nonflipped analysis *							
Local							
S2	R	4813	3.48*E* − 15	5.2	66	−20	26
a.Ins	R			3.5	36	16	4
p.Ins	R			3.58	44	−16	19
IPL	R			4.79	58	−28	28
STG	R			4.31	56	12	4
MTG	R			3.18	56	−50	3
S2	L	4750	4.83*E* − 15	4.64	−62	−22	20
S1	L			2.74	−54	−22	44
a.Ins	L			4.02	−40	−2	−4
S1	R	1671	6.56*E* − 07	−3.78	36	−26	52
cuneus	L	3603	2.55*E* − 12	−4.05	−16	−90	30
Distal							
p.Ins	R	2639	2.6*E* − 10	4.02	34	−18	16
a.Ins	R			4	31	25	−1
IFG	R			2.96	45	13	26
S2	R	1394	2.5*E* − 6	4.47	50	−26	26
STG	R			3.07	57	−37	17
S2	L	3653	4.31*E* − 13	4.41	−58	−26	22
MFG	L			3.0	−50	2	15
SMG	L			3.23	−55	−37	41
IPL	L			4.18	−53	−34	27
a.Ins	L			3.63	−32	20	2
p.Ins	L			3.69	−36	−20	12
Sham							
None							
Verum							
S2	R	7869	2.57*E* − 20	6.53	62	−24	28
SMG	R			4	60	−29	38
STG	R			4.6	60	−38	18
a.Ins	R			4.8	38	22	−2
p.Ins	R			5.3	40	−6	−4
PMC	R			4.8	50	8	40
ITG	R	2633	3.18*E* − 09	−4	44	−70	−6
OCG	R			−3.9	18	−94	28
S1	R	2233	5.96*E* − 08	−4.33	42	−22	50
S1	R			−3.8	22	−28	66
pSMA	R	602	9.15*E* − 03	3.74	4	8	60
SFG	R			3.1	4	24	46
MFG	R			3.95	42	40	12
IFG	R			5.6	56	12	4
MPFC	R	945	4.07*E* − 04	−3.56	4	58	10
Thalamus	R	563	0.0135	3.96	10	−14	8
Thalamus	L			3.79	−10	−18	10
S2	L	7029	9.15*E* − 19	6.34	−62	−28	24
PMC	L			4.4	−55	3	3
SMG	L			5.3	−54	−43	28
STG	L			6.3	−62	−28	24
a.Ins	L			4.83	−34	14	4
p.Ins	L			5.4	−40	0	−4
OCG	L	924	4.87*E* − 04	−3.74	−22	−86	32
*Flipped analysis *							
Local							
S1	R	1885	1.19*E* − 07	−3.84	38	−26	50
S1	L	5566	5.89*E* − 17	3.79	−54	20	43
Distal							
M1	L	3798	1.49*E* − 13	2.51	−52	−6	39
Sham							
None							
Verum							
S1	R	2419	1.02*E* − 08	−4.32	40	−22	48
S1	L	7502	8.89*E* − 20	7.05	−62	−20	22
Thalamus	R	588	0.01	4.77	10	−16	8
Thalamus	L			3.65	−14	−14	8

Note: PMC: premotor cortex, MPFC: medial prefrontal cortex, S1: primary somatosensory cortex, S2: secondary somatosensory cortex, SMG: supramarginal gyrus, SFG: superior frontal gyrus, MFG: middle frontal gyrus, IFG: inferior frontal gyrus, pSMA: presupplementary motor area, STG: superior temporal gyrus, MTG: middle temporal gyrus, ITG: inferior temporal gyrus, a.Ins: anterior insula, p.Ins: posterior insula, OCG: occipital gyrus, IPL: inferior parietal lobe.

Nonflipped analysis: group map with the original orientation of the data as acquired from the scanner. Flipped analysis: subjects with left-sided lesions had their fMRI data flipped across the midsagittal plane to evaluate brain regions known to be lateralized relative to somatosensory stimulation (i.e., S1, M1, thalamus). Cluster size represents the number of voxels in the cluster. “*P* value” represents the cluster probability. “max_*z*” represents normalized probability. “*x*, *y*, *z*” represent the MNI coordinates of the region's peak voxel from the cluster.

**Table 2:** Difference map of brain response to electro-acupuncture.

Region	Side	Cluster size	*P* value	max_*z*		MNI (mm)	
					*X*	*Y*	*Z*
*Nonflipped analysis *							
Local-Sham							
ITG	R	1434	3.0*E* − 5	−3.55	44	−70	−2
IFG	R	574	0.0207	3.63	56	14	4
S2	L	1111	2.61*E* − 4	4.23	−62	−22	20
p.Ins	L		0.0155	2.42	−38	−16	10
S2	R	826	0.00237	4.05	66	−20	26
FIC	R		0.0033	2.94	46	0	6
IPL	R		0.0017	3.13	58	−28	28
STG	R		0.0004	3.56	56	12	4
Distal-sham							
PCC	R	653	0.0102	−3.3	10	−60	18
S2	L	544	0.0273	3.77	−58	−24	18
IPL	L		0.0026	3.01	−52	−34	26
S2	R	489	0.0457	3.46	58	−18	14
IPL	R		0.0065	2.72	52	−34	28
Local-distal							
None							
Verum-sham							
PCC	R	516	0.0354	−3.45	12	−58	14
PCC/precuneus	L	507	0.0385	−3.3	−10	−70	20
Insula	R	879	0.00154	3.38	40	−4	−6
S2	L	1187	1.49*E* − 4	4.43	−60	−22	20
SMG	L		0.0016	3.15	−54	−34	30
STG	L		0.0001	3.98	−62	−28	24
p.Ins	L		0.0083	2.64	−38	−20	8
S2	R	897	0.00134	4.27	62	−18	20
a.Ins	R		0.0045	2.84	30	22	8
*Flipped analysis *							
Local-sham							
S1	R	763	0.00401	−3.46	40	−28	52
Distal-sham							
None							
Local-distal							
None							
Verum-sham							
S1	R	698	0.00698	−3.45	42	−22	48

Note: PCC: posterior cingulate cortex, IFG: inferior frontal gyrus, S1: primary somatosensory cortex, S2: secondary somatosensory cortex, IFG: inferior frontal gyrus, ITG: inferior temporal gyrus, FIC: frontal insula cortex, STG: superior temporal gyrus, MTG: middle temporal gyrus, a.Ins: anterior insula, p.Ins: posterior insula, IPL: inferior parietal lobe. Nonflipped analysis: difference map with the original orientation of the data as acquired from the scanner. Flipped analysis: subjects with left-sided lesions had their fMRI data flipped across the midsagittal plane to evaluate brain regions known to be lateralized relative to somatosensory stimulation (i.e., S1, M1, thalamus). Cluster size represents the number of voxels in the cluster. “*P* value” represents the cluster probability. “max_*z*” represents normalized probability. “*x*, *y*, *z*” represent the MNI coordinates of the region's peak voxel from the cluster.

**Table 3:** Cortical region significantly correlated with change of pain.

Region	Side	Cluster size	*P* value	max_*z*		MNI (mm)	
					*X*	*Y*	*Z*
*Nonflipped analysis *							
Distal							
PFC	R	640	0.00313	−3.36	28	38	20
Verum (local + distal)							
SMA	R/L	788	0.00186	−3.79	0	−30	62
PFC	R	987	0.000342	−3.44	32	48	18
PFC	L	625	0.00826	−3.48	−32	26	38
*Flipped analysis *							
Verum (local + distal)							
S1	R	198	0.0012	−3.46	32	−26	60

Note: S1: primary somatosensory cortex, SMA: supplementary motor area, PFC: prefrontal cortex. Nonflipped analysis: group map with the original orientation of the data as acquired from the scanner. Flipped analysis: subjects with left-sided lesions had their fMRI data flipped across the midsagittal plane to evaluate brain regions known to be lateralized relative to somatosensory stimulation (i.e., S1, M1, thalamus). Cluster size represents the number of voxels in the cluster. “*P* value” represents the cluster probability. “max_*z*” represents normalized probability. “*x*, *y*, *z*” represent the MNI coordinates of the region's peak voxel from the cluster.
